# Emergence, Evolution, and Pathogenicity of Influenza A(H7N4) Virus in Shorebirds in China

**DOI:** 10.1128/JVI.01717-21

**Published:** 2022-02-09

**Authors:** Hongliang Chai, Xiang Li, Minghui Li, Xinru Lv, Wentao Yu, Yi Li, Jing Sun, Yulei Li, Heting Sun, Jingman Tian, Yu Xu, Xiaoli Bai, Peng Peng, Linhong Xie, Siyuan Qin, Qing An, Fengjiang Zhang, Hailong Zhang, Jiang Du, Siyuan Yang, Zhijun Hou, Xiangwei Zeng, Yulong Wang, Juergen A. Richt, Yajun Wang, Yanbing Li, Jianzhang Ma

**Affiliations:** a College of Wildlife and Protected Area, Northeast Forestry University, Harbin, China; b Harbin Veterinary Research Institute, Chinese Academy of Agricultural Sciences, Harbin, China; c General Station for Surveillance of Wildlife Disease & Wildlife Borne Diseases, National Forestry and Grassland Administration, Shenyang, China; d Liaoning Wildlife Protection and Epidemic Disease Monitoring Center, Dalian, China; e Forestry and Grassland Affairs Service Center, Donggang Forestry and Grassland Administration, Donggang, China; f Heilongjiang Vocational College for Nationalities, Harbin, China; g Diagnostic Medicine/Pathobiology, Center of Excellence for Emerging and Zoonotic Animal Diseases (CEEZAD), College of Veterinary Medicine, Kansas State University, Manhattan, Kansas, USA; St. Jude Children's Research Hospital

**Keywords:** AIV, H7N4, shorebird, pathogenicity, mutations, public health

## Abstract

A 2-year surveillance study of influenza A viruses in migratory birds was conducted to understand the subsequent risk during the migratory seasons in Dandong Yalu River Estuary Coastal Wetland National Nature Reserve, Liaoning Province, China, a major stopover site on the East Asian-Australasian flyway. Overall, we isolated 27 influenza A viruses with multiple subtypes, including H3N8 (*n* = 2), H4N6 (*n* = 2), H4N7 (*n* = 2), H7N4 (*n* = 9), H7N7 (*n* = 1), H10N7 (*n* = 7), and H13N6 (*n* = 4). Particularly, a novel reassortant influenza A(H7N4) virus was first identified in a woman and her backyard poultry flock in Jiangsu Province, China, posing a serious threat to public health. Here, we describe the genetic characterization and pathogenicity of the nine influenza A(H7N4) isolates. Phylogenetic analysis indicated that complex viral gene flow occurred among Asian countries. We also demonstrated a similar evolutionary trajectory of the surface genes of the A(H7N4) isolates and Jiangsu human-related A(H7N4) viruses. Our A(H7N4) isolates exhibited differing degrees of virulence in mice, suggesting a potential risk to other mammalian species, including humans. We revealed multiple mutations that might affect viral virulence in mice. Our report highlights the importance and need for the long-term surveillance of avian influenza virus in migratory birds combined with domestic poultry surveillance along migratory routes and flyways and, thereby, the development of measures to manage potential health threats.

**IMPORTANCE** The H7 subtype avian influenza viruses, such as H7N2, H7N3, H7N4, H7N7, and H7N9, were documented as being capable of infecting humans, and the H7 subtype low pathogenicity avian influenza viruses are capable of mutating into highly pathogenic avian influenza; therefore, they pose a serious threat to public health. Here, we investigated the evolutionary history, molecular characteristics, and pathogenicity of shorebird-origin influenza A(H7N4) viruses, showing a similar evolutionary trajectory with Jiangsu human A(H7N4) viruses in HA and NA genes. Moreover, our isolates exhibited variable virulence (including moderate virulence) in mice, suggesting a potential risk to other mammalian species, including humans.

## INTRODUCTION

Similar to the H5 low-pathogenicity avian influenza viruses (LPAIVs), the H7 LPAIVs are capable of mutating into highly pathogenic avian influenza (HPAI) variants that can cause high fatality in poultry and pose a serious threat to public health (1). Since the first human infection with a novel H7N9 subtype avian influenza virus (AIV) in China in the spring of 2013, a total of 1,568 laboratory-confirmed human cases (case fatality rate of 615/1,568, i.e., 39%) have been reported (2). Furthermore, H7N9 HPAI variants emerged in late 2016 and circulated in poultry and humans during the fifth epidemic wave (3). H7N8 and H7N9 HPAI variants were also found in poultry in North America in 2016 and 2017, respectively (4, 5). In summary, the virulence and molecular evolution of influenza A(H7N4) viruses are of great concern to avian and human health.

A novel reassortant A(H7N4) virus was first identified in a woman and her backyard poultry flock in Jiangsu Province, China, in January 2018 (6). Previous studies have shown that partial genes of the human infection-related A(H7N4) viruses were most likely transferred from wild waterfowl to the poultry AIV gene pool (7). Active surveillance of AIVs in migratory birds was undertaken to investigate whether wild birds carry the similar A(H7N4) virus. Consequently, nine H7N4 AIVs were isolated from seabirds in the Dandong Yalu River Estuary Coastal Wetland National Nature Reserve, Liaoning Province; this area is a major stopover site for migratory waterfowl waves on the East Asian-Australasian flyway during migratory seasons.

## RESULTS AND DISCUSSION

Overall, 4,455 samples from shorebirds were collected during the bird migratory season of 2018 to 2019, 27 viruses were isolated, and the total prevalence of AIVs was 0.61%. Viral isolates consisted of multiple subtype combinations, including H3N8 (*n* = 2), H4N6 (*n* = 2), H4N7 (*n* = 2), H7N4 (*n* = 9), H7N7 (*n* = 1), H10N7 (*n* = 7), and H13N6 (*n* = 4) (Table 1). Here, we selected the A(H7N4) viruses isolated in April 2019, which showed a close genetic relationship with the Jiangsu human-related A(H7N4) virus (Js/H7N4), for genetic characterization as part of this investigation (Table 2).

**TABLE 1:** Surveillance of avian influenza virus during the bird migratory season of 2018 to 2019 in Liaoning province, China

Province	Sampling site	Coordinates	Collection date (yr-mo-day)	Sample			Influenza isolate		
				No.	Type	Host	No.	AIV-positive rates (%)	Subtype (no.)
Liaoning	Dandong Yalu River Estuary Coastal Wetland National Nature Reserve	39.83°N, 124.12°E	2018 April 13	979	Fecal dropping	Shorebird	7	0.72	H3N8 (2), H4N6 (2), H4N7 (2), and H7N7 (1)
			2018 October 11	1,273	Fecal dropping	Shorebird	4	0.31	H13N6 (4)
			2019 April 26	1,159	Fecal dropping	Shorebird	16	1.38	H7N4 (9), H10N7 (7)
			2019 September 18	1,044	Fecal dropping	Shorebird	0	0	
**Total**				**4,455**			**27**	**0.61**	

**TABLE 2:** Information about the A(H7N4) isolates

Sampling date (yr-mo-day)	Virus name	Genotype	Host	Isolate ID
2019 April 26	A/eastern curlew/Liaoning/dandong36/2019(H7N4)	1	Eastern curlew (Numenius madagascariensis)	EPI_ISL_445002
2019 April 26	A/eastern curlew/Liaoning/dandong95/2019(H7N4)	1	Eastern curlew (Numenius madagascariensis)	EPI_ISL_445052
2019 April 26	A/shorebird/Liaoning/dandong371/2019(H7N4)	1	Shorebird (species unknown)	EPI_ISL_445090
2019 April 26	A/shorebird/Liaoning/dandong386/2019(H7N4)	1	Shorebird (species unknown)	EPI_ISL_445091
2019 April 26	A/shorebird/Liaoning/dandong603/2019(H7N4)	1	Shorebird (species unknown)	EPI_ISL_445092
2019 April 26	A/shorebird/Liaoning/dandong786/2019(H7N4)	1	Shorebird (species unknown)	EPI_ISL_445093
2019 April 26	A/eastern curlew/Liaoning/dandong688/2019(H7N4)	2	Eastern curlew (Numenius madagascariensis)	EPI_ISL_445051
2019 April 26	A/eastern curlew/Liaoning/dandong1144/2019(H7N4)	3	Eastern curlew (Numenius madagascariensis)	EPI_ISL_445001
2019 April 26	A/little curlew/Liaoning/dandong142/2019(H7N4)	4	Little curlew (Numenius minutus)	EPI_ISL_445089

The whole genomes of Liaoning A(H7N4) (Ln/H7N4) isolates were sequenced and compared with each other. Most gene segments among Ln/H7N4 isolates shared a relatively high nucleotide identity (99.7 to 100%), except for the polymerase acidic (PA), nucleoprotein (NP), and nonstructural (NS) genes, which showed 93.6 to 100%, 93.5 to 100%, and 96.4 to 100% nucleotide identity, respectively. As such, the nine isolates were grouped into four genotypes as follows: genotype 1 [A/shorebird/Liaoning/dandong603/2019(H7N4) (abbreviated as D603)-like, including six isolates], genotype 2 [isolate A/eastern curlew/Liaoning/dandong688/2019(H7N4) (abbreviated as D688)], genotype 3 [isolate A/eastern curlew/Liaoning/dandong1144/2019(H7N4) (abbreviated as D1144)], and genotype 4 [isolate A/little curlew/Liaoning/dandong142/2019(H7N4) (abbreviated as D142)].

**FIG 1:**
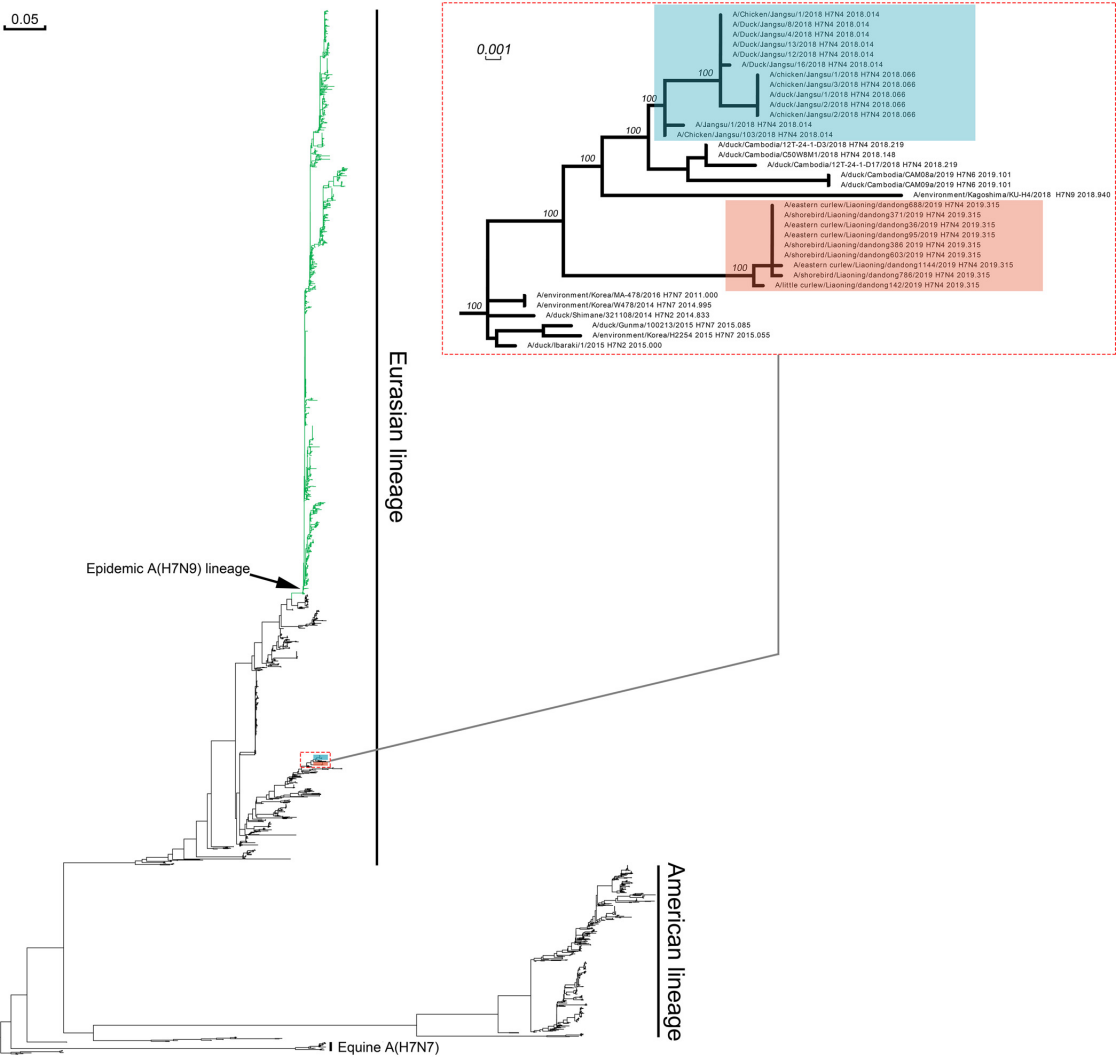
Global phylogenetic history of A(H7) viruses. Red and blue boxes indicate Liaoning shorebird A(H7N4) isolates and Jiangsu human-related A(H7N4) viruses, respectively. Numbers next to main node represent ultrafast bootstrap supports. The branches of epidemic A(H7N9) lineage are colored in green.

**FIG 2:**
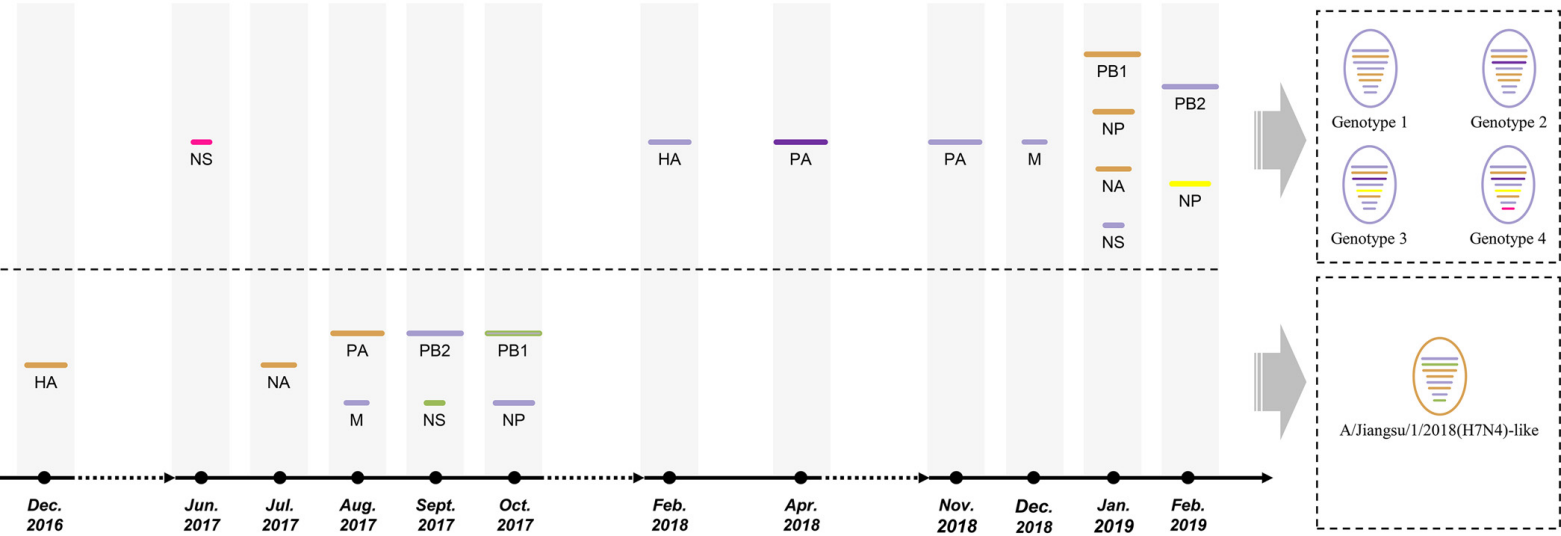
Schematic representation of the origin of Liaoning shorebird A(H7N4) isolates and Jiangsu human-related A(H7N4) viruses. The eight gene segments were (horizontal bars starting from top to bottom of the “virion”) polymerase basic 2, polymerase basic 1, polymerase acidic, hemagglutinin, nucleoprotein, neuraminidase, matrix, and nonstructural. Different colors of gene segments represented different virus origins estimated in the maximum clade credibility trees as follows: purple (A69CCE and 7030A0), yellow (D8A153 and FFFF00), green (969696), and pink (FF1493) represent East Asia, Southeast Asia, China, and Europe, respectively. Timeline indicates the time to the most recent common ancestor (tMRCA).

In the overall genetic structure of the global H7 hemagglutinin (HA) tree, two major geographically dependent genetic lineages, American and Eurasian, were represented. Ln/H7N4 and Js/H7N4 were clustered into the Eurasian lineage; however, they were not genetically related to the enzootic A(H7N9) viruses in China (Fig. 1). The maximum-likelihood phylogenetic inference by the IQ-TREE suggested a similar evolutionary trajectory of the surface genes of Ln/H7N4 and Js/H7N4 (see Fig. S1 in the supplemental material). These results indicate that the HA and neuraminidase (NA) genes of wild waterfowl influenza viruses in China may show common ancestors with Js/H7N4. The six internal genes of A(H7N4) viruses were diverse and clustered into separate lineages, although they mainly clustered with viruses circulating both in wild waterfowl and domestic poultry in the East Asian-Australasian flyway predominantly in Eastern China, Mongolia, Korea, Japan, Cambodia, and Bangladesh (Fig. S1). For Js/H7N4, besides the HA, NA, and PA gene segments, which showed a close relationship with those of A(H7N4) viruses circulating in ducks in Cambodia, the remaining inner gene segments (polymerase basic 1 [PB1], NP, matrix [M], and NS) were clustered together with wild-origin LPAIVs. Compared to the Js/H7N4 that showed common ancestors in all gene segments, Ln/H7N4 showed more diversity in the PA, NP, and NS genes, suggesting that these viruses had undergone multiple reassortment events in viruses circulating in the natural reservoir (Fig. 2; see also Fig. S1). As such, genetic diversity in inner gene segments of the H7N4 virus from shorebird might reflect the breadth of sources from which the AIVs were derived. Our study provided further evidence that highly prevalent reassortment occurred in nature.

**FIG 3:**
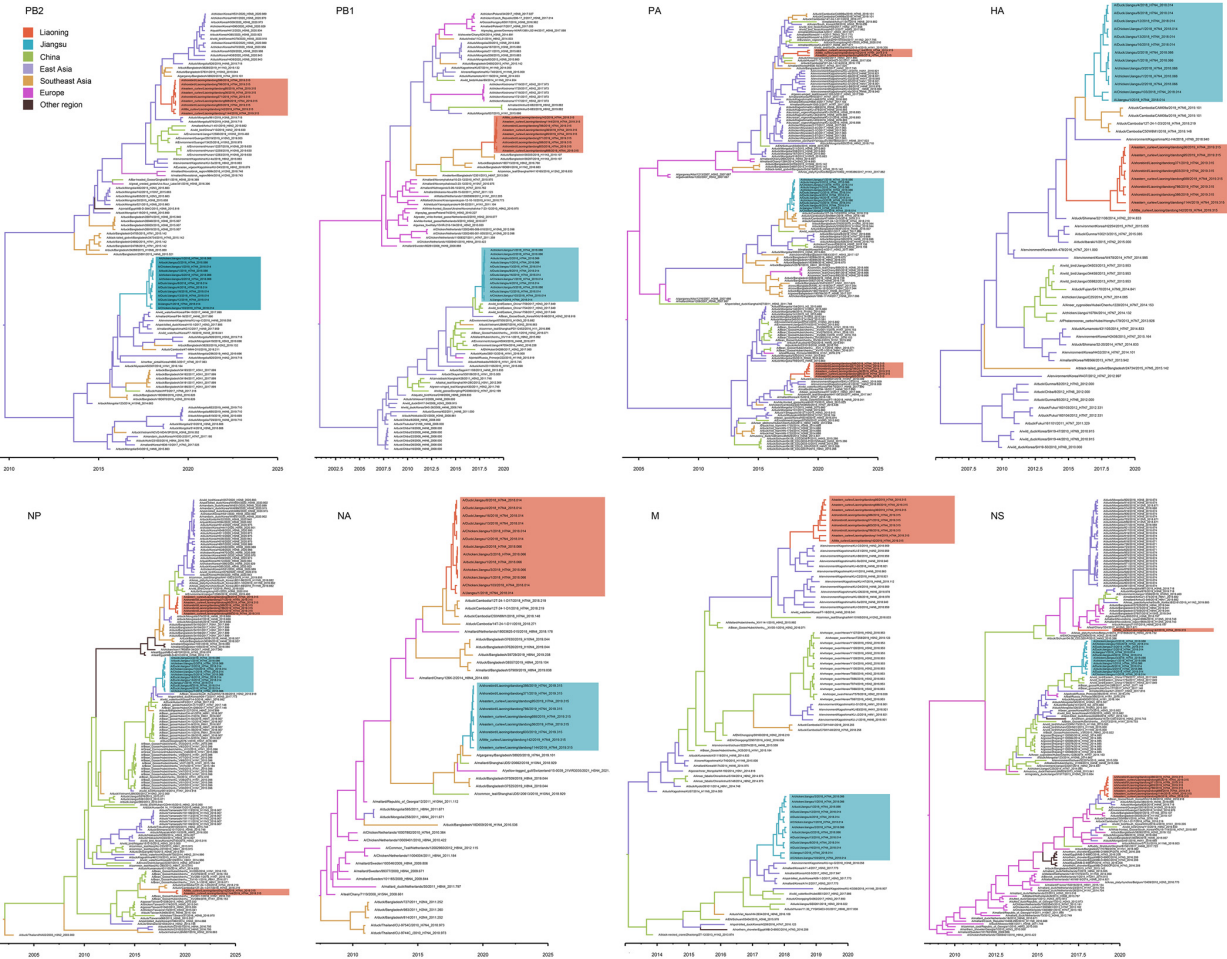
Maximum clade credibility time-scaled phylogenetic tree of the eight segments colored by geographic location. Red and blue boxes indicate Liaoning shorebird A(H7N4) isolates and Jiangsu human-related A(H7N4) viruses, respectively. Segments are shown as follows: polymerase basic (PB2), polymerase basic (PB1), polymerase (PA), hemagglutinin (HA), nucleoprotein (NP), neuraminidase (NA), matrix protein (M), and nonstructural protein (NS).

The timing of evolution events was determined by Bayesian phylogenetic reconstruction and demonstrated by maximum clade credibility trees (Fig. 3). The time to the most recent common ancestor (tMRCA) of the HA and NA genes of Ln/H7N4 was most likely February 2018 and January 2019, respectively, which is later than that of Js/H7N4, which appeared in December 2016 and July 2017, respectively (Fig. 2; Table 3). The tMRCA of the internal genes revealed that the generation of Ln/H7N4 has been a complex process from 2017 to 2019 and was likely completed in February 2019. Reassortment of Js/H7N4 occurred mainly during July to October 2017 (Fig. 2; Table 3).

**TABLE 3:** Bayesian phylogenetic analyses of the eight segments of A(H7N4) virus related to Fig. 2

Segment^*a*^	Correlation coefficient	Cluster (no. of isolates)^*b*^	Origin of most recent common ancestor	tMRCA^*c*^ (mo, yr)	95% HPD^*d*^ interval (mo, yr)	Posterior probability
PB2	0.5443	Jiangsu human A(H7N4)-related strains (13)	East Asia	Sept. 2017	[May. 2017 to Dec. 2017]	1
		Liaoning A(H7N4) isolates (9)	East Asia	Feb. 2019	[Nov. 2018 to Mar. 2019]	1
PB1	0.8696	Jiangsu human A(H7N4)-related strains (13)	China	Oct. 2017	[Jul. 2017 to Dec. 2017]	1
		Liaoning A(H7N4) isolates (9)	Southeast Asia	Jan. 2019	[Oct. 2018 to Apr. 2019]	1
PA	0.8403	Jiangsu human A(H7N4)-related strains (13)	Southeast Asia	Aug. 2017	[Jul. 2019 to Dec. 2019]	0.9994
		Liaoning isolates D142, D688, and D1144	East Asia	Apr. 2018	[Mar. 2018 to Aug. 2019]	1
		Liaoning major A(H7N4) isolates (6)	East Asia	Nov. 2018	[Mar. 2018 to May. 2019]	1
HA	0.9441	Jiangsu human A(H7N4)-related strains (13)	Southeast Asia	Dec. 2016	[Mar. 2016 to Aug. 2017]	0.9883
		Liaoning A(H7N4) isolates (9)	East Asia	Feb. 2018	[Feb. 2017 to Dec. 2018]	1
NP	0.7173	Jiangsu human A(H7N4)-related strains (13)	East Asia	Oct. 2017	[Jul. 2017 to Dec. 2017]	1
		Liaoning isolates D142 and D1144	Southeast Asia	Feb. 2019	[Nov. 2018 to Apr. 2019]	1
		Liaoning major A(H7N4) isolates (7)	Southeast Asia	Jan. 2019	[Oct. 2018 to Mar. 2019]	1
NA	0.9551	Jiangsu human A(H7N4)-related strains (13)	Southeast Asia	Jul. 2017	[Apr. 2017 to Oct. 2017]	0.9998
		Liaoning A(H7N4) isolates (9)	Southeast Asia	Jan. 2019	[Nov. 2018 to Mar. 2019]	1
M	0.7798	Jiangsu human A(H7N4)-related strains (13)	East Asia	Aug. 2017	[May. 2017 to Nov. 2017]	0.9999
		Liaoning A(H7N4) isolates (9)	East Asia	Dec. 2018	[Sept. 2018 to Feb. 2019]	1
NS	0.5865	Jiangsu human A(H7N4)-related strains (13)	China	Sept. 2017	[Jul. 2017 to Nov. 2017]	1
		Liaoning isolate D142	Europe	Jun. 2017	[Mar. 2017 to Sept. 2017]	0.3906
		Liaoning major A(H7N4) isolates (8)	East Asia	Jan. 2019	[Dec. 2018 to Mar. 2019]	1

a PB2, basic polymerase 2; PB1, basic polymerase 1; PA, acidic polymerase; HA, hemagglutinin; NP, nucleoprotein; NA, neuraminidase; M, matrix protein; NS, nonstructural protein.

b D142, A/little curlew/Liaoning/dandong142/2019(H7N4); D688, A/eastern curlew/Liaoning/dandong688/2019(H7N4); D1144, A/eastern curlew/Liaoning/dandong1144/2019(H7N4).

c Time to the most recent common ancestor.

d Highest posterior density.

Using phylogeographic analysis, we sought to understand further the most likely source of A(H7N4) viruses. Potential events of cross-regional gene flow to the origin of A(H7N4) viruses were identified (Fig. 3). For Ln/H7N4, dispersal events from East Asia were observed for polymerase basic 2 (PB2), PA, HA, matrix protein (M), and nonstructural protein (NS) gene; dispersal events from Southeast Asia were detected for PB1, NP, and NA gene. However, intercontinental introductions from Europe were observed in the NS gene of D142, with a low posterior probability of 0.3906. Evidence for gene flow in PB1 and NS genes occurred from Eastern China [A/wildbird/Eastern China/1754/2017(H5N3)-like] to Jiangsu. Viruses in East Asia (PB2, NP, and M) and Southeast Asia (PA, H7, and N4) also contributed to the generation of Js/H7N4. The results indicated that complex viral gene flow occurred among Asian countries. Overlapping of the migration flyway, East Asian-Australasian flyway, might have resulted in this cross-regional dispersal.

The amino acid sequence PELPKGR↓GLFGAI was found within the cleavage site of the HA of Ln/H7N4, suggesting that all were LPAIVs (Table 4). The receptor binding sites were avian-like in 226Q and 228G (H3 numbering) (8). The E627K substitution was observed in the PB2 protein of A/Jiangsu/1/2018; however, the mutation residues Q591, E627, and D701 in the PB2 protein of Ln/H7N4 and Jiangsu poultry A(H7N4) viruses suggest that these viruses have not yet adapted to mammalian hosts (Table 4) (9, 10). Both Ln/H7N4 and Js/H7N4 carried an avian C-terminal ESEV motif in the NS1 protein, which has been shown to increase viral virulence in mice (Table 4) (11).

**TABLE 4:** Key molecular markers of A(H7N4) viruses

Virus	HA (H3 numbering)				NA stalk deletion	PB2				NS1	
	Cleavage site	186	226	228		389	591	627	701	80–84 deletion	PDZ domain
A/little curlew/Liaoning/dandong142/2019(H7N4)	PELPKGR	G	Q	G	No	R	Q	E	D	No	ESEV
A/eastern curlew/Liaoning/dandong1144/2019(H7N4)	PELPKGR	G	Q	G	No	R	Q	E	D	No	ESEV
A/eastern curlew/Liaoning/dandong688/2019(H7N4)	PELPKGR	G	Q	G	No	R	Q	E	D	No	ESEV
A/eastern curlew/Liaoning/dandong36/2019(H7N4)	PELPKGR	G	Q	G	No	R	Q	E	D	No	ESEV
A/eastern curlew/Liaoning/dandong95/2019(H7N4)	PELPKGR	G	Q	G	No	R	Q	E	D	No	ESEV
A/shorebird/Liaoning/dandong371/2019(H7N4)	PELPKGR	G	Q	G	No	R	Q	E	D	No	ESEV
A/shorebird/Liaoning/dandong386/2019(H7N4)	PELPKGR	G	Q	G	No	R	Q	E	D	No	ESEV
A/shorebird/Liaoning/dandong603/2019(H7N4)	PELPKGR	G	Q	G	No	R	Q	E	D	No	ESEV
A/shorebird/Liaoning/dandong786/2019(H7N4)	PELPKGR	G	Q	G	No	R	Q	E	D	No	ESEV
EPI_ISL_376123_A/Jiangsu/1/2018(H7N4)	PELPKGR	G	Q	G	No	R	Q	**K**	D	No	ESEV
EPI_ISL_293286_A/Chicken/Jiangsu/103/2018(H7N4)	PELPKGR	G	Q	G	No	R	Q	E	D	No	ESEV
EPI_ISL_291131_A/Chicken/Jiangsu/1/2018(H7N4)	PELPKGR	G	Q	G	No	R	Q	E	D	No	ESEV
EPI_ISL_332358_A/chicken/Jiangsu/1/2018(H7N4)	PELPKGR	G	Q	G	No	R	Q	E	D	No	ESEV
EPI_ISL_332395_A/chicken/Jiangsu/2/2018(H7N4)	PELPKGR	G	Q	G	No	R	Q	E	D	No	ESEV
EPI_ISL_332396_A/chicken/Jiangsu/3/2018(H7N4)	PELPKGR	G	Q	G	No	R	Q	E	D	No	ESEV
EPI_ISL_332399_A/duck/Jiangsu/1/2018(H7N4)	PELPKGR	G	Q	G	No	R	Q	E	D	No	ESEV
EPI_ISL_293289_A/Duck/Jiangsu/12/2018(H7N4)	PELPKGR	G	Q	G	No	R	Q	E	D	No	ESEV
EPI_ISL_293290_A/Duck/Jiangsu/13/2018(H7N4)	PELPKGR	G	Q	G	No	R	Q	E	D	No	ESEV
EPI_ISL_293291_A/Duck/Jiangsu/16/2018(H7N4)	PELPKGR	G	Q	G	No	R	Q	E	D	No	ESEV
EPI_ISL_332401_A/duck/Jiangsu/2/2018(H7N4)	PELPKGR	G	Q	G	No	R	Q	E	D	No	ESEV
EPI_ISL_293287_A/Duck/Jiangsu/4/2018(H7N4)	PELPKGR	G	Q	G	No	R	Q	E	D	No	ESEV
EPI_ISL_293288_A/Duck/Jiangsu/8/2018(H7N4)	PELPKGR	G	Q	G	No	R	Q	E	D	No	ESEV

**FIG 4:**
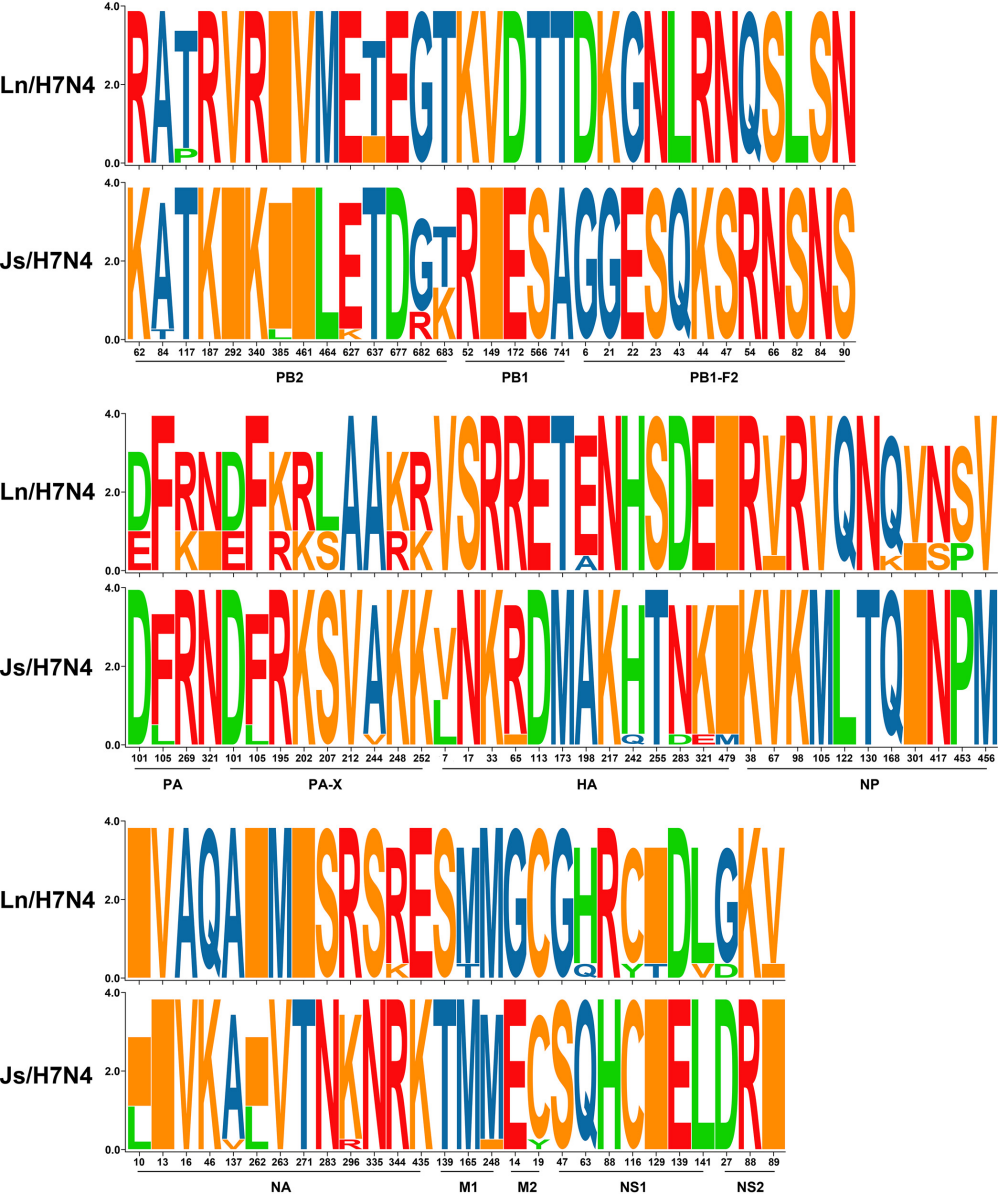
SeqLogo analysis of amino acid substitutions in A(H7N4) viruses. The sequence logo consists of stacks of symbols for corresponding amino acids. Ln/H7N4, Liaoning shorebird A(H7N4) isolates; Js/H7N4, Jiangsu human-related A(H7N4) viruses. Detailed information is available in the supplemental material. See also Table S1 in the supplemental material.

In the SeqLogo analysis (see Table S1 in the supplemental material), Ln/H7N4, especially D1144 and D142, displayed polymorphic amino acid sites in PB2 (*n* = 2), PA (*n* = 3), PA-X (*n* = 6), NP (*n* = 5), NA (*n* = 1), M1 (*n* = 1), NS1 (*n* = 4), and NS2 (*n* = 2). D688 also showed high polymorphic amino acid sites, however, with no single unique site. Virus evolution is characterized by adaptations that are specific to the host. Genetic analysis has shown that Ln/H7N4 and Js/H7N4 shared a common ancestor in HA and NA gene segments; therefore, we next compared their amino acid sequences (Fig. 4; see also Table S1). In particular, A/Jiangsu/1/2018(H7N4) presented three unique amino acids (242Q and 479M in HA and 137V in NA). Five amino acids changed gradually from shorebird to poultry (V7L, D283N, and E321K in HA and I10L and I262L in NA), revealing a potential host adaptation process.

**FIG 5:**
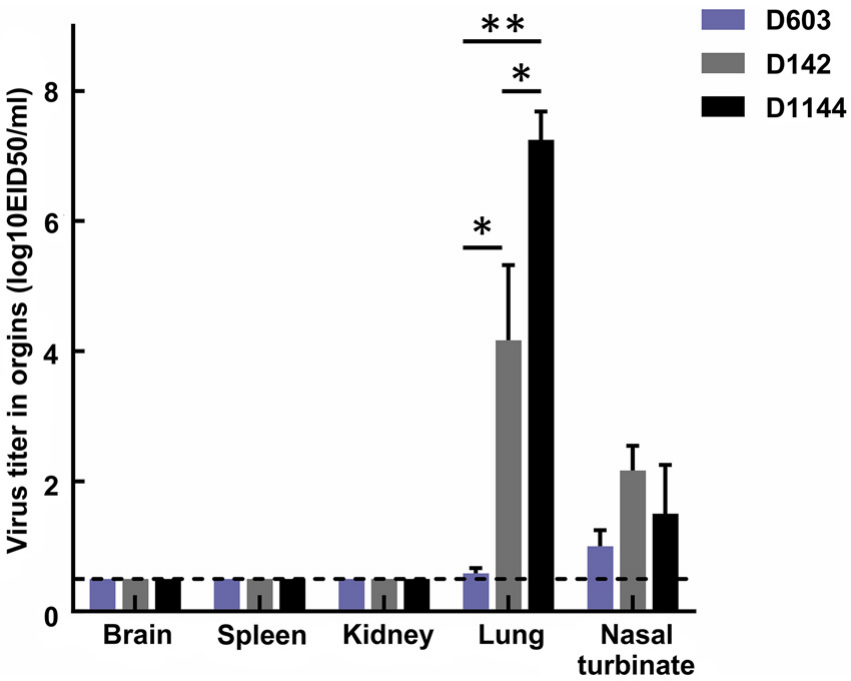
Virus titer in organs (log_10_ EID_50_/ml) collected from mice. Statistical analysis was performed by application of Student's *t* test using GraphPad Prism version 8.0 software. *, *P* < 0.05; **, *P* < 0.01. The error bars represent the SD. D603, A/shorebird/Liaoning/dandong603/2019(H7N4); D142, A/little curlew/Liaoning/dandong142/2019(H7N4); D1144, A/eastern curlew/Liaoning/dandong1144/2019(H7N4).

**FIG 6:**
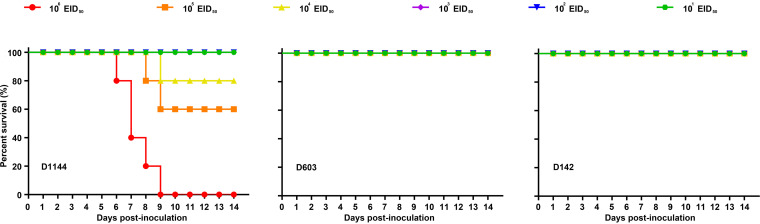
Pathogenicity of the A(H7N4) virus in mice. BALB/c female mice (*n* = 5 per group) were challenged with serial 10-fold dilutions of virus. Survival was observed for 14 days. Mice that lost more than 25% of their body weight and/or became moribund were euthanized.

**FIG 7:**
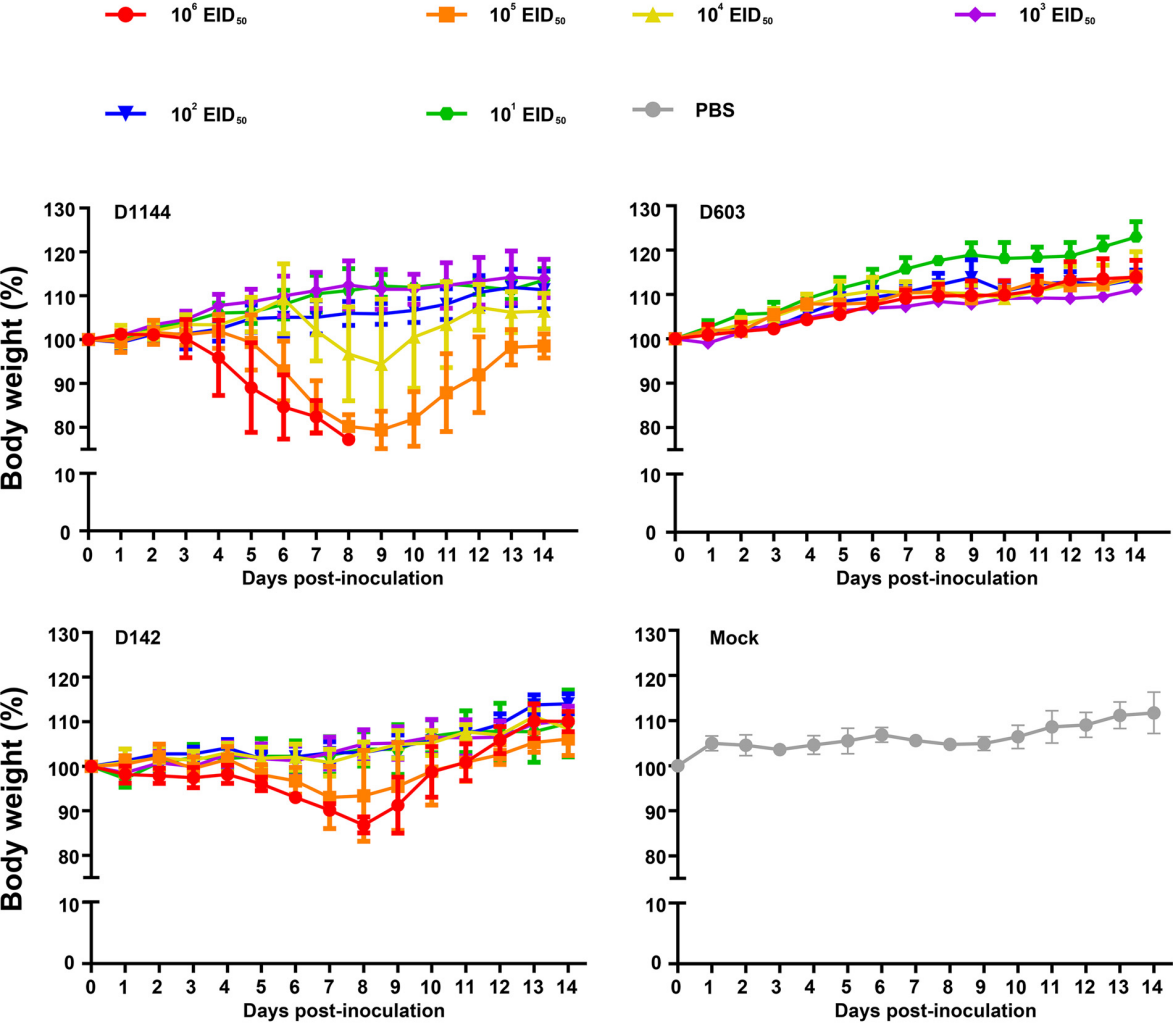
Weight loss in mice infected with the A(H7N4) virus. BALB/c female mice (*n* = 5 per group) were challenged with serial 10-fold dilutions of virus. Body weight was observed for 14 days. Data points indicate mean values and error bars indicate the SD.

**FIG 8:**
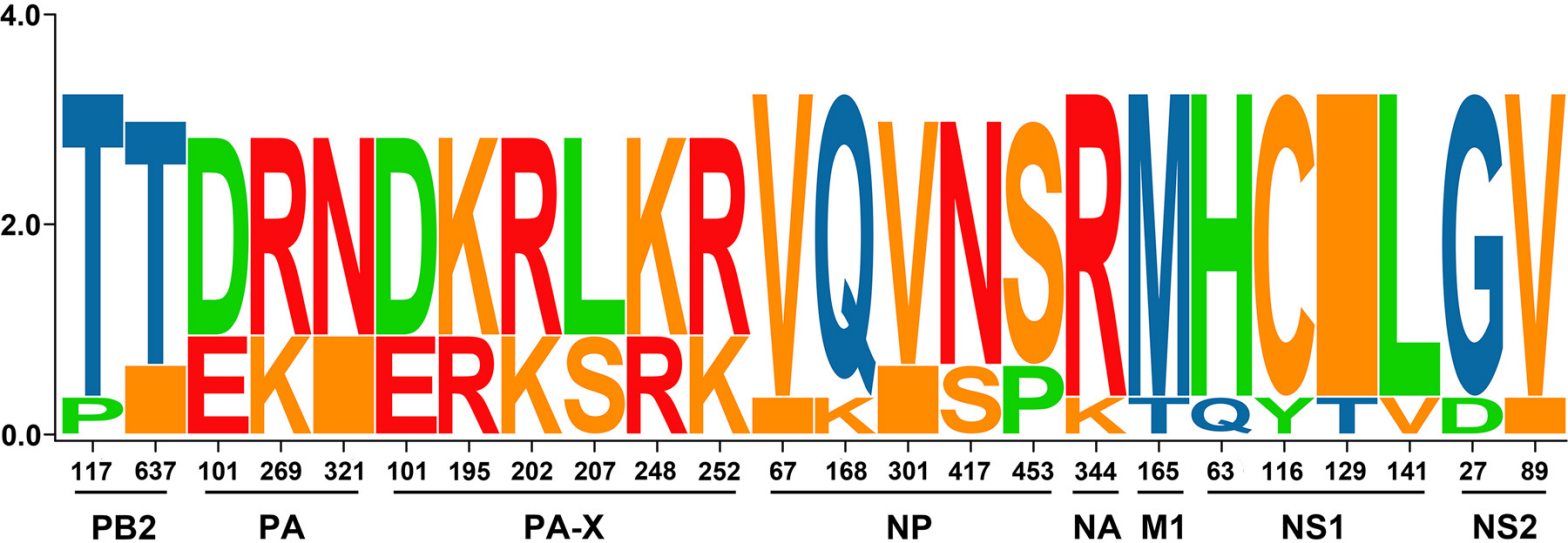
SeqLogo analysis of amino acid substitutions in Liaoning shorebird A(H7N4) isolates. The sequence logo consists of stacks of symbols for corresponding amino acids.

Three viruses were selected and tested by experimental infection of mice to investigate the virulence of the shorebird-origin H7N4 isolates in mammals. Viral titers in organs of D603-infected mice were markedly low (Fig. 5), and all mice survived until 14 days postinfection (dpi) without significant weight loss (Fig. 6 and 7); thus, D603 showed nonpathogenic in mice. In contrast, virus titration showed that D1144 could replicate suitably in the infected mice's lungs (Fig. 5). Furthermore, D1144-infected mice died by day 9 when inoculated with 10^6^ 50% egg infective doses (EID_50_) (Fig. 6). The 50% minimum lethal dose (MLD_50_) of D1144 was 5.43 EID_50_; thus, D1144 exhibits moderate virulence in mice without prior adaption. D142 could be detected in the infected mice's lungs, nasal turbinate, and kidneys (Fig. 5). All D142-infected mice survived until 14 dpi (Fig. 6), although these mice's bodyweight showed a transient decrease from day 4 when inoculated with 10^5^ to 10^6^ EID_50_ and then returned to normal growth by day 12 (Fig. 7). Thus, D142 showed mild virulence in mice. The three viruses exhibited differing degrees of virulence in mice, suggesting a potential risk to other mammalian species, including humans. At the genome level, D1144 and D142 shared similar sources except for the NS gene of D142, which came from Europe. Here, we identified multiple mutations in NS1 (H63Q, C116Y, I129T, and L141V) and NS2 (G27D and V89I), compared to two additional mutations in PB2 (P117T) and NP (V67I) (Fig. 8; see also Table S1). The NS1 protein performs multiple functions that affect AIV replication and virulence (12). Very few NS2 mutations that affect viral fitness and host adaptation are characterized in AIVs. Furthermore, the effect of these mutations on AIV pathogenicity is either negligible or unclear (13). Mutations in PB2 and NP affecting viral virulence have also been confirmed. Thus, mutations H63Q, C116Y, I129T, and L141V in NS1, with P117T in PB2 and V67I in NP might affect viral virulence in mice. These sites potentially affect the biological properties of the virus, but further investigation will be needed. Compared to nonpathogenic strain D603, D1144 only displays one C116Y mutation in NS1; however, multiple mutations were found in other proteins, and experimental verification is required to validate the phenotype of the C116Y mutation NS1.

Live poultry markets have played pivotal roles in the genesis of novel AIVs. In the spring of 2013, a novel A(H7N9) virus emerged in China and infected people exposed to live-bird markets. Phylogenic analysis indicated that the virus emerged through multiple reassortment events in wild birds and obtained internal genes from H9N2 viruses circulating in poultry. H7N9 AIVs were primarily isolated from poultry while occasionally detected in forest bird species. One reasonable explanation for the notable spread of the A(H7N9) virus only in poultry and its geographical limitation to China is that it could not replicate in the waterfowl host, thereby losing the opportunity to infect the migratory birds distributed broadly (14). In this study, we describe the isolation of the A(H7N4) virus from shorebird and their moderate pathogenicity in mice without previous adaption, indicating that these isolates could be a potential threat to the public. Similar A(H7N4) viruses were also detected in Cambodia (15), which provided HA, NA, and PA for the emergence of Js/H7N4. Migratory birds carrying AIVs can travel over long distances. H7N4 subtype LPAIVs also have the possibility of mutating into highly pathogenic strains during transmission or acquire internal genes from A(H9N2) viruses. Given the continual circulation of AIVs in wild birds and domestic poultry, the potential for human spillover, the innate capacity for reassortment of AIVs, and the constant antigenic variation, it is paramount to understand the potential risk of any emerging AIV. Therefore, long-term surveillance of AIVs in migratory birds, combined with domestic poultry surveillance along migratory routes and flyways, should be performed to facilitate early characterization of H7 subtype AIVs and other AIVs.

## MATERIALS AND METHODS

### Sampling, virus isolation, and sequencing.

After the first case of human infection with the A(H7N4) virus was confirmed in January 2018, to trace whether wild birds carry the A(H7N4) virus, active surveillance of AIV was undertaken during the bird migratory seasons of 2018 to 2019 in the Dandong Yalu River Estuary Coastal Wetland National Nature Reserve, Liaoning Province, China. The samples were oscillated and then centrifuged, and the collected supernatant of all samples was inoculated into 9-day-old specific-pathogen-free (SPF) chicken embryos. Then, 72 h after incubation, the allantoic fluid was harvested, and the hemagglutinin (HA) activity was assayed. The negative allantoic fluid was passaged twice again in embryonated eggs. An HA inhibition assay was performed preliminarily to determine the HA subtype of the isolated virus by using chicken anti-HA serum for each subtype. Viral RNA was extracted from HA-positive samples from the incubated allantoic fluid using a QIAamp Viral RNA minikit (Qiagen, Germany) and reverse transcribed using the primer Uni12. Viral cDNAs were amplified by PCR with primers designed for each gene segment (16). The PCR products of eight fragments of the isolates were sequenced using Sanger sequencing. The sequence data were compiled using the SeqMan program (DNASTAR, Madison, WI, USA). The AIV-positive samples’ hosts were identified by amplifying cytochrome *b* sequences as described previously (17). The whole-genome sequences were submitted to the Global Initiative on Sharing All Influenza Data (GISAID) (https://www.gisaid.org/) EpiFlu database (accession numbers EPI_ISL_445001 to EPI_ISL_445093).

### Genetic analysis.

The variation of amino acid sequences among our Liaoning A(H7N4) isolates (abbreviated as Ln/H7N4) and Jiangsu human-related A(H7N4) viruses (abbreviated as Js/H7N4) were visualized by SeqLogo implemented in TBtools to analyze the potential host adaptation molecular characters (18). The sequence logo consists of stacks of symbols for corresponding amino acids. To assess the overall genetic structure of the H7 HA sequences, we downloaded global H7 HA sequences from GISAID. Next, for each gene, Ln/H7N4 and Js/H7N4, together with their top 50 hits in GISAID, were combined into a data set. Sequences were aligned using MAFFT (19) implemented in PhyloSuite 1.21 (20). Duplicated sequences and low-quality sequences were removed. These data sets were further used to infer phylogenies and perform Bayesian analysis. A phylogenetic tree was reconstructed by maximum likelihood (ML) in IQ-TREE (21) under the best-fit substitution model for 10,000 ultrafast bootstraps (22). The best-fit substitution model was selected using the Bayesian information criterion by ModelFinder (23) implemented in PhyloSuite 1.21 (20). Visualization and annotation of the trees were performed by EvolView (https://www.evolgenius.info/evolview-v2).

### Phylogeography analyses.

Before the analysis, the temporal signal of each data set was investigated using a root-to-tip regression of genetic distances against sampling time in the program TempEst v1.5 (24). To clarify the contribution of AIVs in different geographical regions to Ln/H7N4 and Js/H7N4, we performed a phylogeographic analysis using an asymmetric model with a Bayesian stochastic search variable selection implemented in BEAST v1.10.4 (25). The sequences in each data set were divided into multiple geographical regions: Liaoning (Ln/H7N4), Jiangsu (Js/H7N4), China (other Provinces or other subtypes AIVs in China), East Asia, Southeast Asia, Europe, and other region. The best-fit substitution model was selected as described above. We computed the marginal likelihoods using path sampling and stepping-stone sampling (26) to select the best combination of clock model (strict clock and uncorrelated lognormal relaxed clock) and tree priors (constant size, exponential growth, and Bayesian skyline). Two Markov chain Monte Carlo (MCMC) runs with 80,000,000 states sampling every 8,000 steps were performed, and results were combined by LogCombiner v1.10.4 (http://beast.community/logcombiner). Tracer v1.7.1 (http://beast.community/tracer) was used for obtaining an adequate sample size of ≥200. Maximum clade credibility (MCC) trees were combined after removing initial 10% burn-in and then reconstructed using TreeAnnotator v1.10.4 (http://beast.community/treeannotator). MCC trees were edited by FigTree v1.4.3 (http://tree.bio.ed.ac.uk/software/figtree). The time to the most common ancestor was also estimated from the MCC trees. All BEAST runs were run on the cyberinfrastructure for phylogenetic research (CIPRES) science gateway (https://www.phylo.org).

### Ethics statement.

This study was performed according to the recommendations detailed in the Guide for the Care and Use of Laboratory Animals of the Ministry of Science and Technology of the People's Republic of China. The protocol was approved by the Committee on the Ethics of Animal Experiments of the Harbin Veterinary Research Institute, Chinese Academy of Agricultural Sciences.

### Biosafety statement and facility.

Studies with the A(H7N4) viruses were conducted in enhanced animal biosafety level 3 (ABSL3+) facility approved for such use by the Ministry of Agriculture and Rural Affairs of China and China National Accreditation Service for Conformity Assessment. All animal studies were approved by the Review Board of the Harbin Veterinary Research Institute, Chinese Academy of Agricultural Sciences. The facility details and biosafety and biosecurity measures used have been previously reported (27).

### Mouse studies.

Three representative A(H7N4) viruses for genotypes 1, 3, and 4 were used for mouse studies, A/shorebird/Liaoning/dandong603/2019(H7N4), abbreviated as D603; A/eastern curlew/Liaoning/dandong1144/2019(H7N4), abbreviated as D1144; and A/little curlew/Liaoning/dandong142/2019(H7N4), abbreviated as D142. As there is no unique molecular feature, the virus of genotype 2 [A/eastern curlew/Liaoning/dandong688/2019(H7N4), abbreviated as D688] was not selected. Six-week-old female BALB/c mice were obtained from Beijing Vital River Laboratories. For the 50% minimum lethal dose (MLD_50_) testing of the three viruses, groups of five BALB/c mice were anesthetized with CO_2_ and inoculated intranasally with 10-fold serial dilutions 10^6^ to 10^1^ 50% egg infectious doses (EID_50_) of A(H7N4) viruses in a volume of 50 μl. A control group (*n* = 5) was intranasally inoculated with 50 μl of phosphate-buffered saline (PBS). The mice were monitored for 14 days to assess mortality. The MLD_50_ was determined using the Reed-Muench method (28). Three mice were anesthetized with CO_2_ and instilled intranasally with 10^6^ EID_50_ of the virus in a volume of 50 μl to detect the infectivity of A(H7N4) viruses. Three mice were euthanized at 3 days postinfection (dpi), and different tissues, including the brain, spleen, kidney, lungs, and nasal turbinate were collected. The tissue samples were homogenized and centrifuged at 8,000 rpm. Then, the supernatants were collected and inoculated into three 9-day-old eggs. At 72 h after incubation and 37°C, the activity of HA was tested, and the EID_50_ was determined using the Reed-Muench method (28). Statistical analysis was performed by application of Student's *t* test using GraphPad Prism version 8.0 software; *P* < 0.05 was considered as significant. The error bars represent the standard deviation (SD).

### Data availability.

The whole-genome sequences of A(H7N4) isolates in this study were submitted to the Global Initiative on Sharing All Influenza Data (GISAID, https://www.gisaid.org/) EpiFlu database (accession numbers EPI_ISL_445001 to EPI_ISL_445093).
