# Supplementary material for: Emergence, Evolution, and Pathogenicity of Influenza A(H7N4) Virus in Shorebirds in China
Source: J Virol. 2022 Feb 9;96(3):e01717-21. doi: 10.1128/JVI.01717-21 (PMC8826809; doi:10.1128/JVI.01717-21)
Supplement: Supplemental file 1 — Fig. S1 and Table S1. Download JVI.01717-21-s0001.pdf, PDF file, 3.4 MB [file jvi.01717-21-s0001.pdf]

Emergence, Evolution, and Pathogenicity of Influenza A(H7N4) Virus in Shorebirds, China

Supplementary Materials

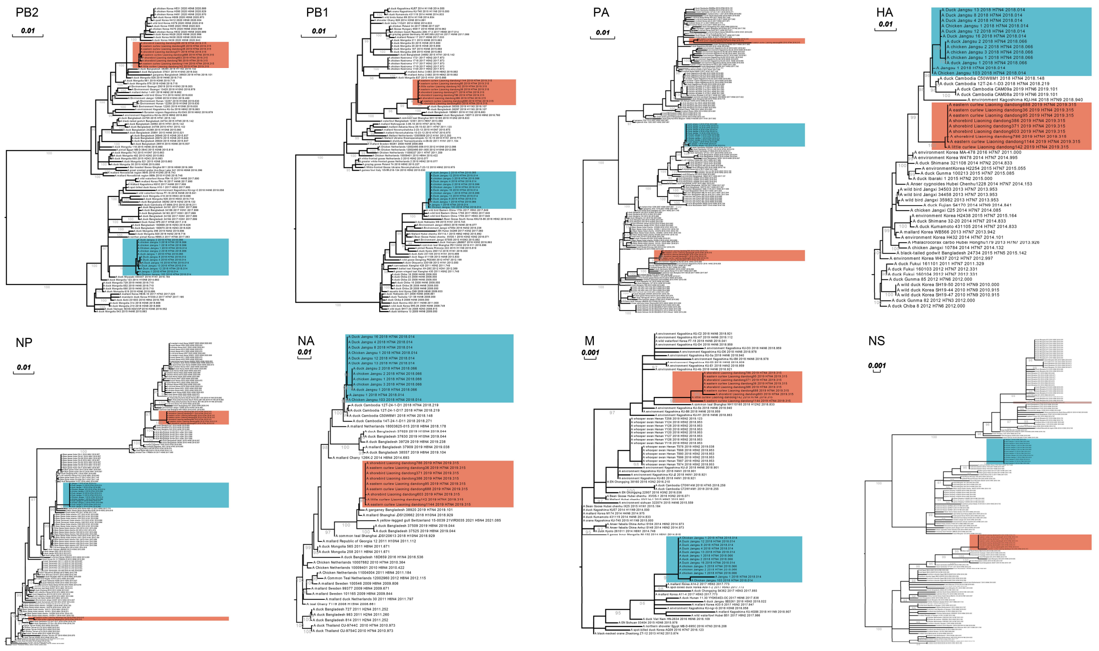

Fig. S1. Maximum-likelihood phylogenetic trees of the eight segments. Red and blue boxes indicate Liaoning shorebird A(H7N4) isolates and Jiangsu human-related A(H7N4) viruses, respectively. Ultrafast bootstrap supports values ≥ 95 were shown. Segments shown are: polymerase basic (PB2); polymerase basic (PB1); polymerase (PA); hemagglutinin (HA); nucleoprotein (NP); neuraminidase (NA); matrix protein (M); nonstructural protein (NS).

Table S1. SeqLogo analysis of amino acid substitutions in A(H7N4) viruses.

Isolate	PB2														PB1					PB1-F2												PA
	62	84	117	187	292	340	385	461	464	627	637	677	682	683	52	149	172	566	741	6	21	22	23	43	44	47	54	66	82	84	90	101
A/little curlew/Liaoning/dandong142/2019(H7N4)	R	A	T	R	V	R	I	V	M	E	T	E	G	T	K	V	D	T	T	D	K	G	N	L	R	N	Q	S	L	S	N	E
A/eastern curlew/Liaoning/dandong1144/2019(H7N4)	R	A	P	R	V	R	I	V	M	E	T	E	G	T	K	V	D	T	T	D	K	G	N	L	R	N	Q	S	L	S	N	E
A/eastern curlew/Liaoning/dandong688/2019(H7N4)	R	A	T	R	V	R	I	V	M	E	I	E	G	T	K	V	D	T	T	D	K	G	N	L	R	N	Q	S	L	S	N	E
A/eastern curlew/Liaoning/dandong36/2019(H7N4)	R	A	T	R	V	R	I	V	M	E	T	E	G	T	K	V	D	T	T	D	K	G	N	L	R	N	Q	S	L	S	N	D
A/eastern curlew/Liaoning/dandong95/2019(H7N4)	R	A	T	R	V	R	I	V	M	E	T	E	G	T	K	V	D	T	T	D	K	G	N	L	R	N	Q	S	L	S	N	D
A/shorebird/Liaoning/dandong371/2019(H7N4)	R	A	T	R	V	R	I	V	M	E	T	E	G	T	K	V	D	T	T	D	K	G	N	L	R	N	Q	S	L	S	N	D
A/shorebird/Liaoning/dandong386/2019(H7N4)	R	A	T	R	V	R	I	V	M	E	T	E	G	T	K	V	D	T	T	D	K	G	N	L	R	N	Q	S	L	S	N	D
A/shorebird/Liaoning/dandong603/2019(H7N4)	R	A	T	R	V	R	I	V	M	E	I	E	G	T	K	V	D	T	T	D	K	G	N	L	R	N	Q	S	L	S	N	D
A/shorebird/Liaoning/dandong786/2019(H7N4)	R	A	T	R	V	R	I	V	M	E	T	E	G	T	K	V	D	T	T	D	K	G	N	L	R	N	Q	S	L	S	N	D
EPI_ISL_376123_A/Jiangsu/1/2018(H7N4)	K	A	T	K	I	K	L	I	L	K	T	D	G	T	R	I	E	S	A	G	G	E	S	Q	K	S	R	N	S	N	S	D
EPI_ISL_293286_A/Chicken/Jiangsu/103/2018(H7N4)	K	A	T	K	I	K	I	I	L	E	T	D	G	K	R	I	E	S	A	G	G	E	S	Q	K	S	R	N	S	N	S	D
EPI_ISL_291131_A/Chicken/Jiangsu/1/2018(H7N4)	K	A	T	K	I	K	I	I	L	E	T	D	G	T	R	I	E	S	A	G	G	E	S	Q	K	S	R	N	S	N	S	D
EPI_ISL_332358_A/chicken/Jiangsu/1/2018(H7N4)	K	A	T	K	I	K	I	I	L	E	T	D	G	T	R	I	E	S	A	G	G	E	S	Q	K	S	R	N	S	N	S	D
EPI_ISL_332395_A/chicken/Jiangsu/2/2018(H7N4)	K	A	T	K	I	K	I	I	L	E	T	D	G	T	R	I	E	S	A	G	G	E	S	Q	K	S	R	N	S	N	S	D
EPI_ISL_332396_A/chicken/Jiangsu/3/2018(H7N4)	K	A	T	K	I	K	I	I	L	E	T	D	G	T	R	I	E	S	A	G	G	E	S	Q	K	S	R	N	S	N	S	D
EPI_ISL_332399_A/duck/Jiangsu/1/2018(H7N4)	K	A	T	K	I	K	I	I	L	E	T	D	G	T	R	I	E	S	A	G	G	E	S	Q	K	S	R	N	S	N	S	D
EPI_ISL_293289_A/Duck/Jiangsu/12/2018(H7N4)	K	T	T	K	I	K	I	I	L	E	T	D	G	K	R	I	E	S	A	G	G	E	S	Q	K	S	R	N	S	N	S	D
EPI_ISL_293290_A/Duck/Jiangsu/13/2018(H7N4)	K	A	T	K	I	K	I	I	L	E	T	D	G	K	R	I	E	S	A	G	G	E	S	Q	K	S	R	N	S	N	S	D
EPI_ISL_293291_A/Duck/Jiangsu/16/2018(H7N4)	K	A	T	K	I	K	I	I	L	E	T	D	R	K	R	I	E	S	A	G	G	E	S	Q	K	S	R	N	S	N	S	D

EPI_ISL_332401_A/duck/Jiangsu/2/2018(H7N4)	K	A	T	K	I	K	I	I	L	E	T	D	G	T	R	I	E	S	A	G	G	E	S	Q	K	S	R	N	S	N	S	D
EPI_ISL_293287_A/Duck/Jiangsu/4/2018(H7N4)	K	A	T	K	I	K	I	I	L	E	T	D	R	K	R	I	E	S	A	G	G	E	S	Q	K	S	R	N	S	N	S	D
EPI_ISL_293288_A/Duck/Jiangsu/8/2018(H7N4)	K	A	T	K	I	K	I	I	L	E	T	D	R	K	R	I	E	S	A	G	G	E	S	Q	K	S	R	N	S	N	S	D
**Isolate**	**PA**			**PA-X**									**HA**													**NP**						
	**105**	**269**	**321**	**101**	**105**	**195**	**202**	**207**	**212**	**244**	**248**	**252**	**7**	**17**	**33**	**65**	**113**	**173**	**198**	**217**	**242**	**255**	**283**	**321**	**479**	**38**	**67**	**98**	**105**	**122**	**130**	**168**
A/little curlew/Liaoning/dandong142/2019(H7N4)	F	K	I	E	F	R	K	S	A	A	R	K	V	S	R	R	E	T	A	N	H	S	D	E	I	R	I	R	V	Q	N	Q
A/eastern curlew/Liaoning/dandong1144/2019(H7N4)	F	K	I	E	F	R	K	S	A	A	R	K	V	S	R	R	E	T	E	N	H	S	D	E	I	R	V	R	V	Q	N	Q
A/eastern curlew/Liaoning/dandong688/2019(H7N4)	F	K	I	E	F	R	K	S	A	A	R	K	V	S	R	R	E	T	E	N	H	S	D	E	I	R	V	R	V	Q	N	Q
A/eastern curlew/Liaoning/dandong36/2019(H7N4)	F	R	N	D	F	K	R	L	A	A	K	R	V	S	R	R	E	T	E	N	H	S	D	E	I	R	V	R	V	Q	N	Q
A/eastern curlew/Liaoning/dandong95/2019(H7N4)	F	R	N	D	F	K	R	L	A	A	K	R	V	S	R	R	E	T	E	N	H	S	D	E	I	R	V	R	V	Q	N	Q
A/shorebird/Liaoning/dandong371/2019(H7N4)	F	R	N	D	F	K	R	L	A	A	K	R	V	S	R	R	E	T	E	N	H	S	D	E	I	R	V	R	V	Q	N	Q
A/shorebird/Liaoning/dandong386/2019(H7N4)	F	R	N	D	F	K	R	L	A	A	K	R	V	S	R	R	E	T	E	N	H	S	D	E	I	R	V	R	V	Q	N	Q
A/shorebird/Liaoning/dandong603/2019(H7N4)	F	R	N	D	F	K	R	L	A	A	K	R	V	S	R	R	E	T	E	N	H	S	D	E	I	R	V	R	V	Q	N	K
A/shorebird/Liaoning/dandong786/2019(H7N4)	F	R	N	D	F	K	R	L	A	A	K	R	V	S	R	R	E	T	E	N	H	S	D	E	I	R	V	R	V	Q	N	Q
EPI_ISL_376123_A/Jiangsu/1/2018(H7N4)	L	R	N	D	L	R	K	S	V	V	K	K	V	N	K	R	D	M	A	K	Q	T	N	K	M	K	V	K	M	L	T	Q
EPI_ISL_293286_A/Chicken/Jiangsu/103/2018(H7N4)	L	R	N	D	L	R	K	S	V	A	K	K	V	N	K	R	D	M	A	K	H	T	D	E	I	K	V	K	M	L	T	Q
EPI_ISL_291131_A/Chicken/Jiangsu/1/2018(H7N4)	F	R	N	D	F	R	K	S	V	A	K	K	V	N	K	R	D	M	A	K	H	T	N	K	I	K	V	K	M	L	T	Q
EPI_ISL_332358_A/chicken/Jiangsu/1/2018(H7N4)	F	R	N	D	F	R	K	S	V	A	K	K	L	N	K	R	D	M	A	K	H	T	N	K	I	K	V	K	M	L	T	Q
EPI_ISL_332395_A/chicken/Jiangsu/2/2018(H7N4)	F	R	N	D	F	R	K	S	V	A	K	K	L	N	K	R	D	M	A	K	H	T	N	K	I	K	V	K	M	L	T	Q
EPI_ISL_332396_A/chicken/Jiangsu/3/2018(H7N4)	F	R	N	D	F	R	K	S	V	A	K	K	L	N	K	R	D	M	A	K	H	T	N	K	I	K	V	K	M	L	T	Q
EPI_ISL_332399_A/duck/Jiangsu/1/2018(H7N4)	F	R	N	D	F	R	K	S	V	A	K	K	L	N	K	R	D	M	A	K	H	T	N	K	I	K	V	K	M	L	T	Q
EPI_ISL_293289_A/Duck/Jiangsu/12/2018(H7N4)	F	R	N	D	F	R	K	S	V	A	K	K	V	N	K	R	D	M	A	K	H	T	N	K	I	K	V	K	M	L	T	Q
EPI_ISL_293290_A/Duck/Jiangsu/13/2018(H7N4)	F	R	N	D	F	R	K	S	V	A	K	K	V	N	K	R	D	M	A	K	H	T	N	K	I	K	V	K	M	L	T	Q
EPI_ISL_293291_A/Duck/Jiangsu/16/2018(H7N4)	F	R	N	D	F	R	K	S	V	A	K	K	V	N	K	I	D	M	A	K	H	T	N	K	I	K	V	K	M	L	T	Q

EPI_ISL_332401_A/duck/Jiangsu/2/2018(H7N4)	F	R	N	D	F	R	K	S	V	A	K	K	L	N	K	R	D	M	A	K	H	T	N	K	I	K	V	K	M	L	T	Q
EPI_ISL_293287_A/Duck/Jiangsu/4/2018(H7N4)	F	R	N	D	F	R	K	S	V	A	K	K	V	N	K	R	D	M	A	K	H	T	N	K	I	K	V	K	M	L	T	Q
EPI_ISL_293288_A/Duck/Jiangsu/8/2018(H7N4)	F	R	N	D	F	R	K	S	V	A	K	K	V	N	K	R	D	M	A	K	H	T	N	K	I	K	V	K	M	L	T	Q
**Isolate**	**NP**				**NA**													**M1**			**M2**		**NS1**								**NS2**	
	**301**	**417**	**453**	**456**	**10**	**13**	**16**	**46**	**137**	**262**	**263**	**271**	**283**	**296**	**335**	**344**	**435**	**139**	**165**	**248**	**14**	**19**	**47**	**63**	**88**	**116**	**129**	**139**	**141**	**27**	**88**	**89**
A/little curlew/Liaoning/dandong142/2019(H7N4)	I	S	P	V	I	V	A	Q	A	I	M	I	S	R	S	R	E	S	M	M	G	C	G	Q	R	C	T	D	V	D	K	I
A/eastern curlew/Liaoning/dandong1144/2019(H7N4)	I	S	P	V	I	V	A	Q	A	I	M	I	S	R	S	R	E	S	M	M	G	C	G	H	R	Y	I	D	L	G	K	V
A/eastern curlew/Liaoning/dandong688/2019(H7N4)	V	N	S	V	I	V	A	Q	A	I	M	I	S	R	S	R	E	S	M	M	G	C	G	H	R	C	I	D	L	G	K	V
A/eastern curlew/Liaoning/dandong36/2019(H7N4)	V	N	S	V	I	V	A	Q	A	I	M	I	S	R	S	R	E	S	M	M	G	C	G	H	R	C	I	D	L	G	K	V
A/eastern curlew/Liaoning/dandong95/2019(H7N4)	V	N	S	V	I	V	A	Q	A	I	M	I	S	R	S	R	E	S	M	M	G	C	G	H	R	C	I	D	L	G	K	V
A/shorebird/Liaoning/dandong371/2019(H7N4)	V	N	S	V	I	V	A	Q	A	I	M	I	S	R	S	R	E	S	M	M	G	C	G	H	R	C	I	D	L	G	K	V
A/shorebird/Liaoning/dandong386/2019(H7N4)	V	N	S	V	I	V	A	Q	A	I	M	I	S	R	S	R	E	S	M	M	G	C	G	H	R	C	I	D	L	G	K	V
A/shorebird/Liaoning/dandong603/2019(H7N4)	V	N	S	V	I	V	A	Q	A	I	M	I	S	R	S	K	E	S	T	M	G	C	G	H	R	C	I	D	L	G	K	V
A/shorebird/Liaoning/dandong786/2019(H7N4)	V	N	S	V	I	V	A	Q	A	I	M	I	S	R	S	R	E	S	M	M	G	C	G	H	R	C	I	D	L	G	K	V
EPI_ISL_376123_A/Jiangsu/1/2018(H7N4)	I	N	P	M	I	I	V	K	V	I	V	T	N	R	N	R	K	T	M	I	E	Y	S	Q	H	C	I	E	L	D	R	I
EPI_ISL_293286_A/Chicken/Jiangsu/103/2018(H7N4)	I	N	P	M	I	I	V	K	A	I	V	T	N	K	N	R	K	T	M	M	E	C	S	Q	H	C	I	E	L	D	R	I
EPI_ISL_291131_A/Chicken/Jiangsu/1/2018(H7N4)	I	N	P	M	I	I	V	K	A	I	V	T	N	K	N	R	K	T	M	M	E	C	S	Q	H	C	I	E	L	D	R	I
EPI_ISL_332358_A/chicken/Jiangsu/1/2018(H7N4)	I	N	P	M	L	I	V	K	A	L	V	T	N	K	N	R	K	T	M	M	E	C	S	Q	H	C	I	E	L	D	R	I
EPI_ISL_332395_A/chicken/Jiangsu/2/2018(H7N4)	I	N	P	M	L	I	V	K	A	L	V	T	N	K	N	R	K	T	M	M	E	C	S	Q	H	C	I	E	L	D	R	I
EPI_ISL_332396_A/chicken/Jiangsu/3/2018(H7N4)	I	N	P	M	L	I	V	K	A	L	V	T	N	K	N	R	K	T	M	M	E	C	S	Q	H	C	I	E	L	D	R	I
EPI_ISL_332399_A/duck/Jiangsu/1/2018(H7N4)	I	N	P	M	L	I	V	K	A	L	V	T	N	K	N	R	K	T	M	M	E	C	S	Q	H	C	I	E	L	D	R	I
EPI_ISL_293289_A/Duck/Jiangsu/12/2018(H7N4)	I	N	P	M	I	I	V	K	A	I	V	T	N	K	N	R	K	T	M	M	E	C	S	Q	H	C	I	E	L	D	R	I
EPI_ISL_293290_A/Duck/Jiangsu/13/2018(H7N4)	I	N	P	M	I	I	V	K	A	I	V	T	N	K	N	R	K	T	M	M	E	C	S	Q	H	C	I	E	L	D	R	I
EPI_ISL_293291_A/Duck/Jiangsu/16/2018(H7N4)	I	N	P	M	I	I	V	K	A	I	V	T	N	K	N	R	K	T	M	M	E	C	S	Q	H	C	I	E	L	D	R	I

EPI_ISL_332401_A/duck/Jiangsu/2/2018(H7N4)	I	N	P	M	L	I	V	K	A	L	V	T	N	K	N	R	K	T	M	M	E	C	S	Q	H	C	I	E	L	D	R	I
EPI_ISL_293287_A/Duck/Jiangsu/4/2018(H7N4)	I	N	P	M	I	I	V	K	A	I	V	T	N	K	N	R	K	T	M	M	E	C	S	Q	H	C	I	E	L	D	R	I
EPI_ISL_293288_A/Duck/Jiangsu/8/2018(H7N4)	I	N	P	M	I	I	V	K	A	I	V	T	N	K	N	R	K	T	M	M	E	C	S	Q	H	C	I	E	L	D	R	I

2
